# Norm-critical elements in nursing and healthcare education: a scoping review

**DOI:** 10.1186/s12909-026-09400-7

**Published:** 2026-05-13

**Authors:** Ivan Andrés Castillo, Kicki Klaeson, Ann-Catrin Ekelund, Ellinor Tengelin

**Affiliations:** 1 Department of Health Sciences, University West, Trollhättan, SE-461 86 Sweden; 2 Medical Library, Region Västra Götaland, Skaraborgs Hospital, Skövde, Sweden; 3 Department of Health Sciences, Occupational Health Science, Mid Sweden University, Östersund, SE-831 25 Sweden

**Keywords:** Healthcare education, Nursing education, Norm-criticism, Norm-critical pedagogy, Health equity, Social justice

## Abstract

**Background:**

Access to healthcare is a fundamental human right that is often influenced by social norms and power structures. Implicit biases in society, healthcare, and education contribute to health inequalities. Incorporating equity and social justice into nursing and healthcare education, through norm-critical pedagogy can empower learners to address and promote social justice and health equity in patient care and community health.

**Methods:**

The aim of the study was to describe characteristics and recurring elements of norm-critical literature in published research. This study followed Arksey and O’Malley’s scoping review framework and adhered to the Preferred Reporting Items for Systematic Reviews and Meta-Analysis extension for scoping reviews to report the findings. A search was conducted in across five scientific databases – CINAHL, APA PsycINFO, ERIC, Medline, and Web of Science – limiting results to English language articles. Two authors independently screened, extracted, and analysed the data, guided by selected key concepts based on the population, concept, and context framework. The included articles were condensed according to specific characteristics and analysed thematically.

**Results:**

Sixteen articles were included in the review, and all published from 2017 onwards, primarily in nursing journals. Most of these articles employed qualitative methodologies. A thematic analysis generated three themes: ‘Redesigning education toward norm-critical pedagogy’, ‘Redefining one’s positionality as a teacher’, and ‘Transforming education for a norm-conscious sustainable healthcare future’.

**Conclusions:**

This scoping review concludes that the characteristics of norm-critical literature, primarily centred on Western nursing education, has limited applicability in other healthcare educations. The literature describes key challenges in integrating norm-criticism into educational structures, often placing undue responsibility on individual teachers to adopt a norm-critical pedagogy. This review calls for further empirical research on norm-criticism to advance its use as a pedagogical model in nursing and healthcare education.

**Supplementary Information:**

The online version contains supplementary material available at 10.1186/s12909-026-09400-7.

## Background

Access to healthcare is an essential human right [1]. However, the access to healthcare is often influenced by social norms and institutionalised power structures [2]. Despite this, persistent health inequalities continue to impact individuals and communities, with long-lasting effects for health and well-being [3]. In the healthcare sector, nurses make up the largest occupational group [4] and research suggests that normative assumptions and implicit biases in nursing education may contribute to these inequalities [4]. For instance, a qualitative study of gender expectations among nursing students [5] and an analysis of structural racism and implicit biases in nursing education [6] illustrates how normative gender and racial assumptions become structurally embedded in nursing education, and in turn, shape health equity outcomes.

To address this, nursing and healthcare education must equip future professionals with the ability to critically analyse how power, privilege, and societal norms shape patient care. Incorporating knowledge of equity and social justice into nursing curricula is increasingly recognized as a pedagogic necessity, as it can empower nursing students to address health equity as health professionals [7]. Healthcare programmes, in fields such as medicine, physical therapy, and occupational therapy have also highlighted the importance of incorporating health equity and social justice knowledge into their curricula [8–11]. In this endeavour, norm-criticism has been described as a valuable pedagogical strategy, grounded in a critical framework that interrogates dominant norms and aims to make visible the mechanism of exclusion and marginalisation in different settings [12]. Over the past two decades, norm-critical pedagogy has gained significance in Nordic countries [13]. Although norm-criticism has its foundation in feminist, queer, and post-structural traditions that theorise normality as a regulatory effect of discourses and power, norm-criticism focuses on norm production rather on individual bias or tolerance [14]. Drawing on the post-structural concept of problematisation, norm-critical pedagogy can be understood as a pedagogical practice that interrogates how certain identities or experiences come to be constituted as ‘problems’ through social and institutional practices, and how such problem representations simultaneously establish norms about what counts as normal and deviant, respectively [15]. In this sense, norm-critical pedagogy does not primarily seek to correct prejudiced individuals or empower marginalised groups, but to make visible and critically examine the normative frameworks through which problems and subjects are produced. However, unlike post-structural and feminist analytical problematisation or critical pedagogy, norm-criticism operationalisations of this analytical stance pedagogically by redirecting attention from the oppressed or the oppressor to the norms that enables oppression, presenting pedagogical methods to map, displace, and redesign normative practices to avoid replacing one exclusionary norm with another [16].

In Western nursing education, norm-critical pedagogy has been suggested as a teaching strategy to highlight health equity and enable nursing students to reflect on health inequalities. This pedagogy prepares students to address these disparities and reduce the power imbalance between nurses and patients [17]. The pedagogy involves reflecting on and problematising the privileges and powers held by individuals and groups deemed ‘normal’ (i.e., within the societal norm) and examining the impact of these privileges on those perceived as ‘other’ (i.e., those not regarded as ‘normal’) [17]. In this way, students are encouraged to reflect on and discuss their own roles as future caregivers while becoming aware of how ‘normalisation’ and ‘othering’ may affect patient care [18].

Despite the growing attention to norm-critical pedagogy in healthcare educational discourse, the body of peer-reviewed research in this field remains fragmented. Initial searches across databases, such as CINAHL and MEDLINE showed no existing literature reviews, scoping reviews, or systematic reviews on this topic. Therefore, this scoping review intends to systematically map how norm-critical pedagogy has been conceptualised and integrated into healthcare education. Further, given the relative novelty of norm-criticism as a pedagogy and the absence of comprehensive overviews, a scoping review can provide a valuable synthesis of existing literature, identify knowledge gaps, and outline future research.

## Methods

### Aim

This scoping review aimed to describe research literature on nursing and healthcare education that explicitly is defined as norm-critical. Specifically, we aimed to describe:

1. characteristics of the published research, and
2. recurring elements in the included norm-critical literature.

### Study design

Scoping reviews are suitable for providing an overview of emerging research topics and evaluating their size and scope [19]. This scoping review was guided by Arksey and O’Malley’s [20] 5-step methodological approach, supported by the population, concept, and context (PCC) structure [21], to ensure the search strategy aligned with the study’s aim. The Preferred Reporting Items for Systematic Reviews and Meta-Analysis extension for scoping reviews (PRISMA-ScR) was followed [22].

#### Phase one: identifying concepts related to the aim

The first step involved identifying the key concepts most closely related to the topic under study [20]. The initial mapping allowed us to determine the central ideas that would guide the review. These concepts were then iteratively refined and specified so they could be clearly formulated and utilised within the PCC structure (Table 1).

**Table 1:** Concepts developed by following the PCC structure

Population	Concept	Context
Nursing students, nursing teachers	Norm-criticism	Nursing education or healthcare education

#### Phase two: identifying relevant publications

To refine the search strategy, index terms were chosen to complement the PCC concepts, as displayed in Table 2. Terms such as ‘norm-awareness’ and ‘norm-creativity’ were included as index terms because they are sometimes used synonymously with norm-criticism. This strategy was chosen to scope literature that explicitly conceptualised itself in relation to norm-criticism, rather than to capture all norm-challenging pedagogical approaches. In May 2025, a research librarian conducted systematic searches using key concepts and index terms across five scientific electronic databases: CINAHL, APA PsycINFO, ERIC, Medline, and Web of Science. No restrictions were placed on study design, publication type or publication date, albeit only English-language peer-reviewed publications were included. Grey literature was excluded, in favour of published peer-reviewed primary literature. By not including grey literature the expectation was to enhance the transparency and reproducibility of the systematic search, as grey literature is not as always as accessible or has the same standard of vetting process as peer-reviewed literature. I.A.C and K.K conducted additional manual searches of reference lists of the articles to identify any relevant literature not captured by the systematic search. This process resulted in 316 records, with 145 duplicates removed, leaving 171 records for screening.

**Table 2:** Concept, context and index terms used in database searching

Concept	Searched in: Keywords/title/abstract/index terms
Norm-criticism	(‘norm* criti*’ OR ‘norm* aware*’ OR ‘norm* creativ*) AND
Context	Searched in: Keywords/title/abstract/index terms
Nursing Education / Healthcare Education	((nurs* or health*) AND (educat* or school* or learn* or teach* or classroom* or universit* or academic))

#### Phase three: study selection

**Fig. 1:**
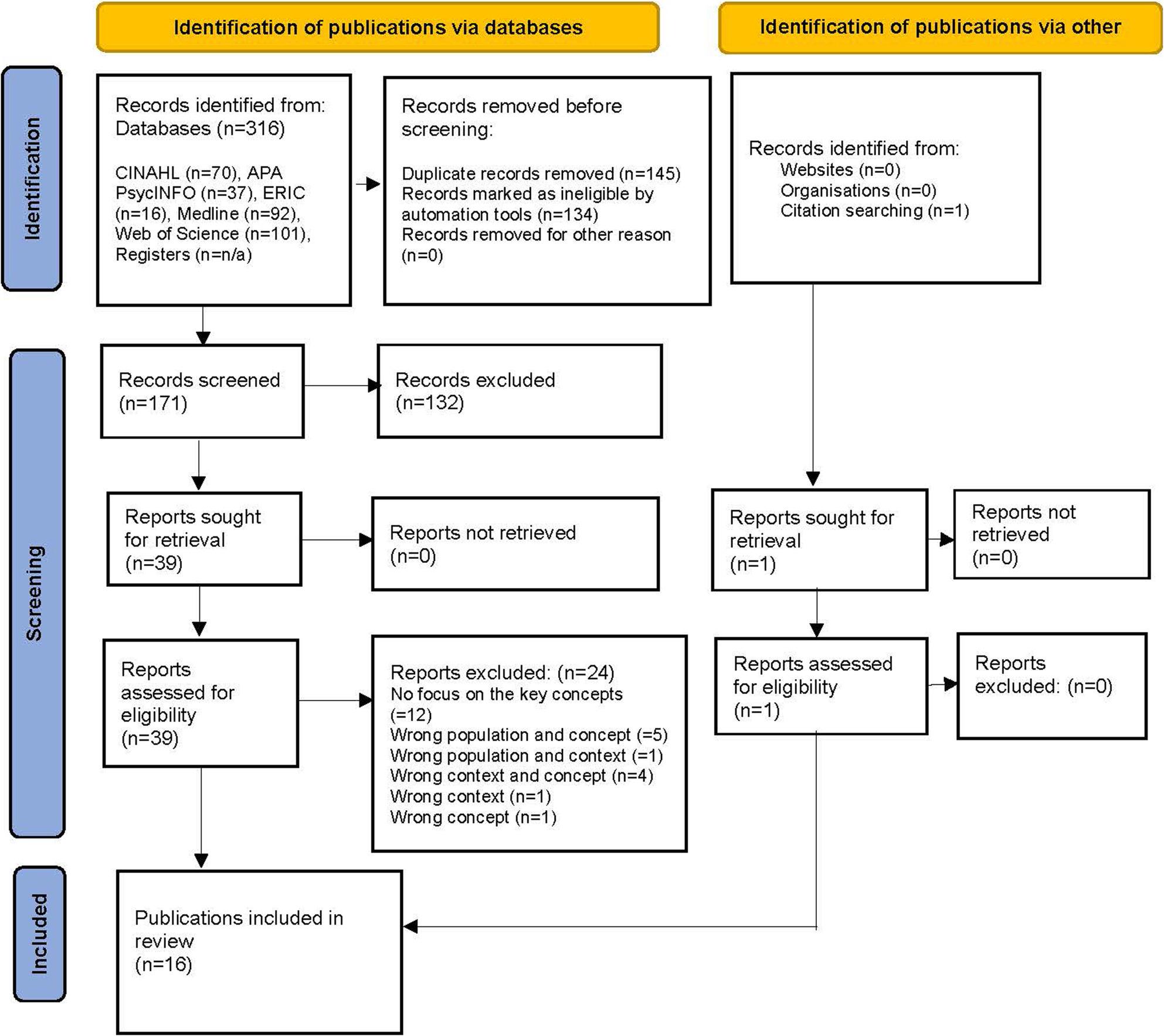
Study selection process – PRISMA-ScR chart

I.A.C and K.K independently screened titles and abstracts of the 171 publications, which involved reading the abstract and evaluating them based on the PCC concepts (Table 1.) 132 publications were excluded. The remaining 39 publications were retrieved in full-text format and assessed for eligibility independently by I.A.C and K.K. The eligibility criterion was that all PCC concepts had to be included in the publication. Following author discussions, 24 publications were excluded, leaving 15 for the final review. One additional publication was included after manually searching the reference lists of the assessed publications, resulting in 16 publications for review. All 16 publications were peer-reviewed scientific journal articles. The list of included and excluded articles, including full-text articles, is shown in the PRISMA-ScR flowchart (Fig. 1.)

#### Phase four: charting the data

In the fourth stage, I.A.C and K.K independently chartered the data from the included articles [20]. The data were extracted and organised, including author(s), year of publication, country, journal name, article title, keywords, aim, research design, and results into and Excel spreadsheet; then visualised in a summery table (Table 3).

**Table 3:** Summary of articles that met the inclusion criteria

Author(s) Year of publication Country of origin	Journal	Title of research paper	Aim/purpose	Research design	Result/ Findings
Arveklev and Tengelin (2024) [23] Sweden	Nurse Education Today	Learning to teach at a norm-critical learning centre: A phenomenographic study	To describe nursing teachers’ conceptions of learning norm-critical approaches and implementing them in a campus-based clinical learning center.	Qualitative phenomenographic study, Interviews	This study showed that teachers who are facing the task of providing norm-critical, practical education to nursing students at CBCLEs are ambivalent towards the core principles of norm-criticism, which they consider natural and provoking, at the same time.
Castillo et al. (2023) [24] Sweden	Nursing Inquiry	When nursing education becomes political: Norm-critical perspectives in a campus-based clinical learning environment	This case study aimed to gain an understanding of and elaborate on an educational development initiative in which norm criticism was incorporated into the composition of a new campus-based clinical learning environment for nursing education.	Qualitative case study, Interviews and documents	Three themes were generated: ‘Intention to educate beyond nursing education’, ‘Educating in alliance with society’, and ‘The educative ambiguity of the Clinical Learning Centre’
Castillo et al. (2025) [25] Sweden	Journal of Contemporary Ethnography	Norm-critical teaching in practice? An observational study of a campus-based clinical learning environment	To identify how nursing teachers approach norm-criticism during learning activities in a campus-based clinical learning environment with a norm-critical profile.	Qualitative participatory observational study, fieldnotes	The study identified that the teachers did not know how or lacked the tools to use norm-critical teaching strategies, although small traces of norm-critical conceptualization could be observed throughout the observations. Ultimately, the norm-critical perspective in the CBCLE appeared to be interlinked to particular teachers’ ability or and willingness to engage with the perspective but also to insufficient structural support to do so.
Dillard-Wright and Nye (2024) [26] United States of America	Journal of Nursing Education	Queering the poster: Disruptive knowledge at the AACCN diversity symposium	To conceptualize what queering academic nursing spaces might look like using a poster we presented as a case study	Academic article, Conceptualisation	The structure of the expanded poster is replicable across different kinds of nursing educational spaces and topics …//… Inviting learners into this kind of co-created, ongoing educational activity upends the hierarchies of conference participation, breaking the fourth wall. This kind of work also has potential for the classroom, whether in physical or virtual space.
Holmgren (2017) [27] Sweden	Nordic Journal of Nursing Research	Global nursing: Educating future nurses for tomorrow’s nursing care needs	The question is, are future nurses educated with enough relevant knowledge and skills to be able to meet tomorrow’s nursing care needs?	Academic debate article, Conceptualisation	Global nursing is about developing knowledge about health, care, persons, suffering and ecology in local and global contexts. The subject profile area is characterised by advocacy, activism and sustainable development. This includes the active use of knowledge and methods that include norm- critical approaches to counteract inequalities and social injustice.
Iheduru-Anderson and Wahi (2022) [28] United States of America	The Online Journal of Issues in Nursing	Race and racism discourse in U.S. nursing: Challenging the silence	To deconstruct how language to address racism in nursing has been used historically; explain why using this language has not been successful; and describe innovative approaches to racial discourse to directly address racism in healthcare and nursing education.	Qualitative, Conceptualisation	The article advocates for teaching norm-critical thinking and discourse, developing norm-critical skills, to nurse faculty and students. Thus, empowering the nursing profession to recognise and dismantle institutionalised racism.
Mitchell et al. (2021) [29] Canada	Nursing Inquiry	Writing activities and the hidden curriculum in nursing	To look for markers in the interviews that pointed to how hidden curriculum power structures and objectivist values.	Qualitative, Interviews	Faculty can counteract the impact of the hidden curriculum on writing activities by loosening the reins on assignment structure, acknowledging power relationships, and using strength-based approaches to reward gains in nursing discourse.
Nye et al. (2023) [30] United States of America	Nursing Inquiry	Exposing othering in nursing education praxis	Define othering and examine how it manifests in nursing education.	Qualitative, Conceptualisation	Norm criticism is proposed as a powerful theoretical framework through which to examine nursing education praxis and disrupt the embedded mechanisms of ‘othering’. This disruption work is collective, collaborative, and continual, with no fixed set of metrics or endpoints. Rather than disheartening, this reality is exciting; collective action holds the greatest possibility for reimagining nursing education praxis that resists ‘othering’ and promotes equity.
Nye and Dillard-Wright (2023) [31] United States of America	Journal of Nursing Education	Queering the classroom: teaching nurses against oppression.	Attempt to model queer norm-criticism and lay the theoretical foundation for transformation of nursing education praxis.	Qualitative, Conceptualisation	This article serves as a call to action for nursing educators, inviting them into critical reflexivity by offering a queered lens through which to dismantle oppression within the practice and praxis of nursing education.
Nye et al. (2023) [32] United States of America	Advances in Nursing Science	Developing a theory of norm-criticism in nursing education	Explore the core concepts of norm-critical pedagogy developed in Sweden and only recently applied to nursing education praxis.	Qualitative, Conceptualisation	The article presents the development of a theoretical model within the field of norm-critical pedagogy …//… The model has three key characteristics, norms which refer to societal expectations, rules and standards that influence behaviors and interactions. Power entails how power structures impact teaching, learning, and ‘othering’, which is a process of defining and excluding individuals and groups based on perceived differences. These three characteristics interact with each other in nursing education, resembling interlocking gears in a clock. By interrupting this clock with structurally oriented critical reflexivity different systematic and structural inequalities can be dismantled and a meaningful change of oppressive structures in nursing education can commence.
Pelters (2018) [33] Sweden	International Journal of Qualitative Studies on Health & Well-Being	On mountains and prophets: targeting majorities to support minorities by using norm-critics in health education	To suggest and describe another way of dealing with the stereotypic expectations accompanying and creating stigma by targeting its normative basis.	Academic debate article, Conceptualisation	Norm-critical education is a way of making members of the (presumably healthy) normative majority uncover and question their health-related norms. …//…It is concluded that norm-critics render a valuable and much needed addition to the health intervention repertoire, especially in the field of health disparities which to date has mostly focused on coping and applying pedagogy of tolerance.
Razack and Philibert (2019) [34] Canada	Medical teacher	Inclusion in the clinical learning environment: Building the conditions for diverse human flourishing	Explore how inclusion, diversity, and equity can and should be defined and operationalized within medical education.	Qualitative, case analysis that observes teachers and medical students	Inclusive CLEs provide space for co-creation, understand the need to ensure the voices of the vulnerable (i.e. learners) are heard and valued and through this promote the flourishing of diverse human capital, in keeping with a model that views diversity as a key attribute of organisational excellence.
Tengelin et al. (2019) [35] Sweden	Advances in Nursing Science	Norm-critical potential in undergraduate nursing education curricula: A document analysis.	To explore whether and how norm-critical perspectives are reflected in the formal documents and literature of nursing education.	Qualitative case study, Documents	The analysis shows that nursing education appears to be guided by certain underlying norms, conventional rather than critical, despite the rhetoric that exists in places in the guiding documents. One of these norms appears to be that the nursing profession is an individual rather than a societal concern.
Tengelin et al. (2019) [36] Sweden	Equality, Diversity and Inclusion	Constructing the norm-critical awareness scale: A scale for use in educational contexts promoting awareness of prejudice, discrimination, and marginalisation	To develop a scale for measuring norm-critical awareness.	Mixed methods, instrument development	The results indicate that in our sample of nursing students, the theoretically derived dimensions of function, consequences, identity, resistance and learning measure the underlying construct of norm-critical awareness and that a summary NCAS score does indeed reflect this construct.
Tengelin et al. (2020) [37] Sweden	Nursing Inquiry	From political correctness to reflexivity: A norm-critical perspective on nursing education	To explore constructions of norms and normality among students in nursing education.	Qualitative, Open-ended survey questions	We could see how the students’ constructed norms and normality as (a) instrumental instructions, consisting of easy-to-digest statements grounded in the profession’s obvious matter of reflection, with each individual being responsible for understanding differences in norms, perspectives and opinions.
Tengelin and Dahlborg-Lyckhage (2017) [38] Sweden	Nursing Inquiry	Discourses with potential to disrupt traditional nursing education: Nursing teachers’ talk about norm-critical competence	To describe the discourses underlying nursing teachers’ talk about their own norm-critical competence.	Qualitative, Interviews	Three discourses were identified in their talk, all of which had the potential to disrupt traditional, normative nursing education.

#### Phase five: collating, summarising, and reporting the results

In this phase, to answer study aim (a), we collated and condensed the chartered data. We identified and highlighted characteristic features of the included articles and presented them in a clear and accessible manner [19], including a description of the keywords used in the articles.

To answer study aim (b), the results of the articles were analysed and thematised [20] inspired by Braun and Clarke’s [39] reflexive thematic analysis to describe norm-critical elements in nursing and healthcare education. Because the aim of the scoping review was to map patterns across a body of literature, the analysis was conducted at a semantic level, focusing on the explicit meanings communicated in the articles included. This approach enabled the identification of shared patterns across literature while enabling a conceptual synthesis. The thematic analysis process involved dividing the text into data extracts, coding, clustering codes, formulating subthemes, reviewing and revising subthemes, and generating overarching themes. A critical appraisal of the methodological quality of the included studies was not conducted, as the purpose of the scoping review was to map existing literature and identify knowledge gaps regarding recurring norm-critical elements in nursing and healthcare education.

## Results

### Characteristics of included articles

The publication dates of the articles spanned from 2017 to 2025 and were geographically concentrated in Western societies. Nine articles originated from Sweden [23–25, 27, 33, 35–38] whereas the remaining articles were from North America [26, 28–32, 34]. Twelve articles were published in nursing journals [23–24, 26–32, 35, 37, 38], one in a medical journal [35], one in a health science journal [33], and two in social sciences and humanities journals [25, 36].

The most prevalent methodological approach was qualitative (*n* = 12), with seven articles classified as theoretical conceptualisations [26–28, 30–33] and four [23, 24, 29, 38] as interview studies. Other methodological approaches included two academic debate articles [27, 33], one observational article [25], one case analysis [34], one mix-method article [36], and a survey article [37].

Only six articles included specific participants. In two articles [36, 37], nursing students participated by completing surveys (*n* = 363 in total across both articles), while in one article [29], the nursing students participated in face-to-face interviews (*n* = 20). Other specified participants included faculty members from nursing education programmes: academic nurse educators (*n* = 20 in total) [23, 38] and executive faculty staff (*n* = 7) [24].

### Thematic analysis

The thematic analysis of the included articles generated three themes and six subthemes.

#### Theme one: redesigning education toward norm-critical pedagogy

This analysis showed that a key element of norm-criticism was the strategy of altering or expanding the current structure of nursing or healthcare education, such as learning environments and curricula^24^–^25^, ^27^–^31^, ^33^–^34^. The first subtheme, ‘*Molding the learning environments*’ describes how to integrate norm-critical pedagogy into clinical learning environments. Learning environments are essential to nursing and healthcare education and are highlighted as physical spaces that promote critical consciousness, inclusivity, equity, and social justice in healthcare. By altering the composition of the clinical learning environments – both through norm-critical art and by co-creating learning spaces with students – students and teachers alike can be encouraged to challenge existing hierarchies, norms, and biases, thereby developing norm-critical skills. The second subtheme, ‘*Expanding the curricula*’ highlighted the need to incorporate norm-critical pedagogy into current curricula. The underlying assumption of expanding the curricula was that there is a predominant focus on teaching and learning for standardisation, which limits teaching and learning in the areas of inclusiveness, equity, and social justice. Therefore, to balance the curricula, norm-criticism was presented as a robust theoretical pedagogical framework that can be effective in teaching, learning, and subsequently, in knowledge production regarding power imbalances, ‘othering’, and biases in nursing and healthcare education:

*Therefore*,* rather than considering a deconstruction and disruption of oppressive structures and systems to be additive*,* more—more material*,* more courses*,* and more learning outcomes to measure—norm-criticism allows us instead to question and disrupt established norms and practices with the aim of creating a truly antioppressive teaching praxis. *(Extract from Nye and Dillard-Wright [31], p. 194)

Norm-critical pedagogy has also been highlighted as an alternative to tolerance pedagogy because of the normative manner in which tolerance pedagogy counteracts biases, power imbalances, and equity. In contrast to tolerance pedagogy, norm-critical pedagogy is presented as a form of teaching and learning about one’s own norms, privileges, powers, and relationships with others within a healthcare context. Although the reviewed articles focused on the healthcare context, there was an underlying direction in the curricula alterations that transcended beyond nursing and healthcare, thus connecting to a global discussion on issues of societal inclusion, equity, and justice.

#### Theme two: redefining one’s own positionality as a teacher

The analysis also emphasised that teachers’ learning processes and positioning were key elements of norm-critical pedagogy. The focus on teachers’ personal attitudes toward the development of teaching has been highlighted in Arveklev and Tengelin [23], Castillo et al. [25], Iheduru-Anderson and Wahi [28], Nye et al. [30], Nye and Dillard-Wright [31], Nye et al. [32], Razack and Philibert [34], Tengelin et al. [35], Tengelin et al. [37], and Tengelin and Dahlborg-Lyckhage [38]. The subtheme, ‘*Challenging one’s own position as a teacher’*, described the necessity for teachers to critically examine their own perspective on ‘othering’ and power, and how these shape structural inequalities in clinical practice and education. Norm-critical pedagogy was presented as a vital tool for teachers to enable them to expand their own teaching practices. By applying a norm-critical lens to one’s teaching materials, teachers could modify non-inclusive or oppressive content to be more inclusive and support a socially just curriculum. For example, teachers could identify binary sections in their teaching materials, such as using the terms ‘mother’ or ‘dad’ instead of ‘legal guardian’:

*…Norm-criticism was talked about in relation to the teachers’ own personal learning processes. The teachers stressed the value of learning*,* but also the insufficiency of their new knowledge. One metaphor used was that of being ‘equipped*,*’ suggesting that strong student reactions were to be expected if the norm-critical perspective were implemented in teaching. *(Extract from Tengelin and Dahlborg-Lyckhage [37], p. 6)

The second subtheme *‘A personal re-learning process’*, centres on the teachers’ transformative learning processes of acknowledging their personal norms, power, and positions, and how they relate to the concept of ‘othering’. By employing norm-criticism in their own practices and reflecting on ‘othering’, teachers learn how to teach norm-criticism and inclusion. The articles highlight that by using self-reflection, as proposed by norm-critical pedagogy, teachers critically examine their perspectives, positions, or teaching as teachers and how their personal privileges may influence their teaching. This personal relearning can foster a norm-critical awareness in teachers, which could influence their teaching to be more centred on inclusiveness, equity, and social justice, and consequently, on students’ understanding of care.

#### Theme three: transforming education for a norm-conscious sustainable healthcare future

The analysis outlines norm-critical pedagogy as a key element in holistic and socially attuned nursing and healthcare education. Norm-criticism was promoted as a pedagogy with the potential to stimulate students to become norm-aware healthcare practitioners, who can make profound social changes concerning inclusiveness, equity, and social change in the future [24, 26, 28, 30, 32–33, 37]. In subtheme, ‘*Fostering students to be norm-aware*’, norm-critical awareness was identified as an essential skill for students within the healthcare context to develop. Although students were not the primary focus of the reviewed articles, norm-critical pedagogy and learning were upheld as an indirect path to cultivate critical thinking, self-reflectivity, and norm-awareness among students. By applying norm-criticism in teaching, students would be equipped to recognise and challenge societal norms, which ultimately foster equitable, inclusive, and socially just healthcare practices. Norm-critical pedagogy was also recognised as a catalyst for social change, as highlighted in the subtheme ‘*Adapting education for social change*’. The reviewed articles emphasised that incorporating norm-criticism into the educational context and engaging in a self-reflective process of analysing one’s own norms and privileges would enable students, teachers, and faculty to challenge and transform educational and healthcare norms that inhibit inclusiveness, equity, and social justice. Ultimately, norm-critical pedagogy was presented as an educational mission to foster critical understanding and awareness of power imbalances in societal structures, thus preparing future professionals to drive social change while navigating complex professional environments and societies:

*Norm-critical education is a way of making members of the (presumably healthy) normative majority uncover and question their health-related norms. *(Extract from Pelters [33], p 9).

## Discussion

This review aimed to describe characteristics and recurring norm-critical elements in published research. The findings show that research literature on norm-criticism within nursing and healthcare education is predominantly concentrated in nursing education fields, particularly in Western countries. The concentration of norm-critical literature in these regions can be traced to the historical development. Originating from critical, queer, and feminist pedagogies, norm-criticism has evolved in Nordic countries since 2010 [13, 40], which explains its growing in Western countries.

Another explanation could be the institutionalised norms that steer the process of planning, conducting, and publishing research. Horton [41] argued that because the science and public health concerns of the developed world are the norm, researchers and editors internalise these norms, often neglecting diverse research perspectives and unconsciously reinforcing research and publishing biases. In a study by Nielsen et al. [42], two high-impact-factor journals in psychology showed that between 2006 and 2010, 96% of the articles had a first author from a Western country, and 90,5% of the participants shared similar cultural backgrounds as the authors. Therefore, expressions of norm-criticism as a pedagogy in education worldwide may not ‘fit’ the academic or scientific discourse used in scientific journals. This potentially limits the full scope of what norm-critical pedagogy in education could encompass. However, it should be noted that this scoping review only encompasses peer-reviewed research literature, excluding governmental documents, legislation, or national curricular guidelines. These kinds of policy documents may signal and encourage movement towards more inclusive educational models. Thus, this scoping review does not provide the whole picture of norm-critical pedagogy in educational practice.

One of the key norm-critical elements found was the redesign of education by shaping learning environments and expanding curricula. In this process, essential concepts such as norms, power, privileges, and ‘othering’ were highlighted as fundamental in defining norm-critical pedagogy. Despite the emphasis on norm-critical pedagogy, the articles reveal gaps regarding the differences between redesigning education within the framework of norm-critical pedagogy versus other pedagogical models. Most articles acknowledge that norm-criticism is rooted in critical pedagogy, referring to foundational works by Kumashiro [43] and Freire [44]. While several articles are theoretically inclined, they provide limited descriptions of the differences and similarities between norm-critical and critical pedagogy. This is also reflected in the essential concepts used in norm-critical pedagogy (i.e., norms, power, privileges, etc.), which are similar to those used in critical pedagogy. For example, Kumashiro [43] elaborated on concepts such as oppression, ‘othering’, privileges, and power in critical pedagogy contexts. Another question that arises concerning the essential concepts of norm-critical pedagogy is how they relate to other frameworks used in nursing and healthcare education to address teaching and learning on issues of equity and social justice, such as decolonisation [45, 46] and intersectionality [47, 48]. Thus, the findings of our scoping review suggest that for norm-critical pedagogy to advance in the redesign of education, it would benefit from disentangling its essential concepts, clarifying how they relate to other pedagogical models and highlighting its uniqueness; namely, the focus on how norms are discursive produced, sustained, and pedagogically enacted in everyday practices [14]. This focus distinguishes norm-critical pedagogy form critical pedagogy, which places greater emphasis on structural power oppressions and emancipation through the development of critical thinking.

Another element found in this review was the emphasis on teachers’ ability to learn and relearn to become norm-critical teachers. Teachers were presented as learners. Björck and Willermark [49] emphasised that work-integrated learning is a multidimensional learning phenomenon that includes professionals’ learning in their working practice. Work-integrated learning has been described as a transitional process where one’s personal and professional lives intersect, learning experiences in the workplace are reflected upon, and the professional self is redefined [50]. In the articles reviewed, norm-critical pedagogy was also linked to teachers’ abilities to transform themselves through self-reflection on their own powers, privileges, and conceptions to advance their teaching for equity and social justice. The findings revealed that norm-critical elements in nursing and healthcare education included teachers redefining their roles as educators through relearning and transition. However, this could be interpreted as placing excessive responsibility on each teacher to undergo such a personal journey. At the same time, such expectations may also reflect broader institutional conditions in contemporary higher education, where institutional change is often pursued through the reflective and self-transformative work of individuals [51]. Moreover, it should also be acknowledged that there are other organisational and structural norms, such as professional standards, faculty board hierarchies, and societal actors that socialises teachers to take on expected roles [52, 53]. The individual emphasis observed in the reviewed literature may therefore not necessarily be inherent to norm-critical pedagogy but may also be shaped by now educational change is organised and governed within higher education systems [54]. This disproportionate responsibility for teachers themselves was moderately elaborated upon in the reviewed articles, and there was no specific mention or explanation of *how* to achieve this transformation, exempt from self-reflection. Consequently, our scoping review indicates that for norm-critical pedagogy to advance in nursing and healthcare education, there is a need to prioritise and support teachers with norm-critical pedagogical tools. Developing such tools, and embedding them within institutional and curricular structures, may help shift the focus from individual transformation alone toward more sustainable pedagogical practices.

A third central norm-critical element is the direction toward the future, making education more inclusive, socially attuned, and sustainable. The American Association of Colleges of Nursing [55] pointed out that health equity and social justice are crucial elements in nursing education, and De Sousa et al. [56] indicated that nursing students find social justice perspectives in their education valuable. The call to incorporate perspectives on inclusiveness, equity, and social justice into nursing and healthcare education has lingered for some time. Voices have been raised that education should equip students with tools that promote health equity and social justice early on in students’ education, to give future practitioners abilities to ensure holistic and qualitative care [57, 58]. Numerous research highlight health equity and social justice as integral components of nursing education and healthcare education [8, 59, 60]. However, these concepts are not prioritised in curricula, and their understanding varies across geographic regions [8]. Canales and Drevdahl [61] maintained that health equity and social justice are treated as rhetorical concepts in nursing education rather than being genuinely prioritised. These concepts frequently receive insufficient attention because students and nurses are socialised to deliver care exclusively in the nurse–patient relationship, grounded in the biomedical model of healthcare, which excludes a broader focus on community care [62]. Our findings indicate that a key norm-critical element in the literature was an educational mission that emphasised the impact of societal norms impact on healthcare and patient experiences, which extended beyond the nurse–patient relationships. Central to norm-critical pedagogy is the need for nursing and healthcare to understand the impact of norms, whether in a profession, education, or practice. Bell [63] underlined that nursing education has become politically safe, that is, in compliance with the reinforcement of socially dominant norms of classism, heteronormativity, racism, and sexism. By dismantling norms and power hierarchies through teaching and learning, nursing education can disrupt the current status [64, 65] and advance toward becoming a more socially attuned education. Thus, our findings indicate that central in norm-critical pedagogy is its claim to potentially develop into a robust educational model in which knowledge about inclusiveness, equity, and social justice can be harboured, making a substantial contribution to future education.

However, despite the focus of norm-critical pedagogy on advancing socially attuned nursing and healthcare education, it appears from this scoping review that norm-critical pedagogy is predominantly articulated through theoretical conceptualisations; there were few empirical articles of norm-critical pedagogical practices. Theoretical articulations are however not just rhetorical; they constitute forms of problematisations through which norms and educational practices are made thinkable and governable [15]. While theoretical and discursive articulations thus play a productive role in shaping the field, empirical knowledge of norm-critical pedagogical practice remain limited. The interplay between theoretical knowledge and practical knowledge is pivotal for nursing and healthcare disciplines [66]. Therefore, a future direction for norm-critical research should be to explore norm-criticism as a praxis. Additionally, the implications of this scoping review indicate that incorporating norm-critical pedagogy in nursing and healthcare education requires structural as well as pedagogical shifts. The identified norm-critical elements highlight opportunities for curriculum innovations. Advancing norm-critical pedagogy in nursing and healthcare education therefore demands that pedagogical strategies are developed and integrated within broader educational frameworks.

## Limitations

This scoping review was limited to peer-reviewed English-language literature, and we acknowledge that limiting the scoping review to a specific language may have potentially overlooked other relevant research in other languages on norm-criticism. This restriction may introduce language bias, reduce generalisability of our findings, and potentially omit important insights presented in non-English articles. Additionally, a systematic search of grey literature was not conducted, hence, the review may potentially exclude practice-based reports and other literature resources of potential relevance. Critical appraisal was not omitted, as it is not a requirement for scoping reviews [19], which may have affected somewhat the study’s quality. A review protocol was not registered for this scoping review, as protocol registration is not required under the Arksey and O’Malley [19] framework; however, the review followed this framework and its subsequent refinements to ensure methodological rigor. Owing to time and resource constraints, stakeholder involvement in the review process, as recommended by Arksey and O’Malley [19], was not possible. An additional limitation relates to the composition of the included literature. Several of the articles were authored by individuals in the research group. This reflects, in part, the narrow and term-specific search strategy used, which centred on norm-criticism as it is conceptualised within nursing education. Our own positionality and sustained involvement in norm-critical nursing education research may likewise have shaped both the research process, how we identified, and selected eligible articles. This includes our familiarity with specific conceptual traditions, scholarly communities, and publications pathways which may have inadvertently contributed to the greater visibility of research areas already known to us. Moreover, our methodological choices have influenced the conclusions of this scoping review by limiting possible analysis of the ontological and epistemological assumptions underpinning norm-critical pedagogy in nursing and healthcare education. Our search strategy may have privileged conceptual and theoretical articulations of the field. The limited number of empirical articles should therefore not necessarily be interpreted as evidence that norm-critical praxis is absent; it may indicate that such praxis has not yet been operationalised within research designs that our scoping review was not designed to capture. Thus, future scoping reviews in would benefit from expanded search terms and conceptual linkages to more fully capture how norm-challenging pedagogies are theorised, developed, enacted, and studied across diverse global and educational settings.

## Conclusion

To the best of our knowledge, this is the first scoping review to focus on norm-critical elements in nursing and health education. This study aimed to describe characteristics and recurring elements of norm-criticism in published research literature. The review showed that norm-critical literature predominantly focuses on nursing education, with limited contributions from other healthcare educational fields. It also highlighted that this literature is primarily situated in Western contexts and is dominated by theoretical, qualitative conceptualisations. Advancing a norm-critical pedagogical practice in nursing and healthcare education requires further empirical research to explore its application and implications in practice.

The review also identified three key norm-critical elements in the included norm-critical literature: redesigning education toward norm-critical pedagogy, redefining teachers’ positionality, and transforming education for a norm-conscious sustainable healthcare. However, the findings also revealed knowledge gaps that must be addressed to advance norm-critical pedagogy in nursing and healthcare education, such as distinguishing norm-critical pedagogy from other pedagogical frameworks, particularly critical pedagogy. The literature highlights the necessity for teachers continuously learn and relearn to become norm-critical educators. This may place an undue responsibility on individual teachers. Further research is also required on effectively integrating norm-critical pedagogy into broader educational structures.

Norm-critical pedagogy is presented as an educational model that promotes inclusiveness, equity, social justice, and future-oriented social attunement. Yet, the review highlights that norm-critical pedagogy remains largely underdeveloped. This limitation presents an opportunity to advance norm-critical research by exploring its application in practice and examining it as a praxis–integrating theoretical and practical knowledge. Although norm-criticism and its pedagogy is a young concept, it offers a novel perspective in nursing and healthcare education. Our study reveals critical knowledge gaps that must be addressed for norm-critical pedagogy to progress and remain relevant within these fields.

## Supplementary Information

Supplementary Material 1.

## Data Availability

All data generated and analysed during this study are included in the published article.

## Abbreviations

PCC: Population, Concept, and Context
PRISMA-ScR: Preferred Reporting Items for Systematic Reviews and Meta-Analysis extension for scoping reviews
