# Supplementary material for: Norm-critical elements in nursing and healthcare education: a scoping review
Source: BMC Med Educ. 2026 May 13;26:759. doi: 10.1186/s12909-026-09400-7 (PMC13169698; doi:10.1186/s12909-026-09400-7)
Supplement: Supplementary file 1 — Supplementary Material 1. [file 12909_2026_9400_MOESM1_ESM.docx]

Appendix Search terms

CINAHL, EBSCO

| **#** | **Query** |
| --- | --- |
| S5 | S1 AND S2 AND S3 Limiters - Language: Danish, English, Norwegian, Swedish |
| S4 | S1 AND S2 AND S3 |
| S3 | TI ( nurs* or health* ) OR  AB ( nurs* or health* ) OR  SU ( nurs* or health* ) OR  MW ( nurs* or health* ) |
| S2 | TI ( educat* or school* or learn* or teach* or classroom* or universit* or academic ) OR  AB ( educat* or school* or learn* or teach* or classroom* or universit* or academic ) ) OR  SU ( educat* or school* or learn* or teach* or classroom* or universit* or academic ) OR  MW ( educat* or school* or learn* or teach* or classroom* or universit* or academic ) |
| S1 | norm* N1 criti* OR norm* N1 aware* OR norm* N1 creativ* |

APA PsycINFO, EBSCO

| **#** | **Query** |
| --- | --- |
| S5 | S1 AND S2 AND S3 Limiters - Language: Danish, English, Norwegian, Swedish; Publication Type: All Journals |
| S4 | S1 AND S2 AND S3 |
| S3 | TI ( nurs* or health* ) OR AB ( nurs* or health* ) OR SU ( nurs* or health* ) OR MJ ( nurs* or health* ) |
| S2 | TI ( educat* or school* or learn* or teach* or classroom* or universit* or academic ) OR  AB ( educat* or school* or learn* or teach* or classroom* or universit* or academic ) OR  SU ( educat* or school* or learn* or teach* or classroom* or universit* or academic ) OR  MJ ( educat* or school* or learn* or teach* or classroom* or universit* or academic ) |
| S1 | norm* N1 criti* OR norm* N1 aware* OR norm* N1 creativ* |

ERIC, EBSCO

| **#** | **Query** |
| --- | --- |
| S5 | S1 AND S2 AND S3  Limiters - Publication Type: Journal Articles; Language: Danish, English, Swedish |
| S4 | S1 AND S2 AND S3 |
| S3 | TI ( nurs* or health* ) OR AB ( nurs* or health* ) OR SU ( nurs* or health* ) |
| S2 | TI ( educat* or school* or learn* or teach* or classroom* or universit* or academic ) OR AB ( educat* or school* or learn* or teach* or classroom* or universit* or academic ) OR SU ( educat* or school* or learn* or teach* or classroom* or universit* or academic ) |
| S1 | norm* N1 criti* OR norm* N1 aware* OR norm* N1 creativ* |

Medline (ALL), OVID

| \|  \| **Searches** \| \| --- \| --- \| \| 1 \| ((norm* adj2 criti*) or (norm* adj2 aware*) or (norm* adj2 creativ*)).ab,hw,kf,ti. \| \| 2 \| (educat* or school* or learn* or teach* or classroom* or universit* or academic).ab,hw,kf,ti. \| \| 3 \| (nurs* or health*).ab,hw,kf,ti. \| \| 4 \| 1 and 2 and 3 \| \| 5 \| limit 4 to (danish or english or norwegian or swedish) \| |
| --- | --- | --- | --- | --- | --- | --- | --- | --- | --- | --- | --- | --- |

Web of Science, Clarivate

| TS= (Norm* NEAR/1 criti*) or (Norm* NEAR/1 aware*) or (Norm* NEAR/1 creativ*) |
| --- |
| AND |
| TS= (educat* or school* or learn* or teach* or classroom* or universit* or academic) |
| AND |
| TS= (nurs* or health*) |
| AND |
| English (Languages) |
| AND |
| Article or Review Article or Early Access (Document Types) |
